# Manipulating the cGAS-STING Axis: advancing innovative strategies for osteosarcoma therapeutics

**DOI:** 10.3389/fimmu.2025.1539396

**Published:** 2025-02-07

**Authors:** BingBing Li, Cheng Zhang, XiaoJuan Xu, QiQin Shen, ShuNan Luo, JunFeng Hu

**Affiliations:** ^1^ Department of Pediatrics, Shaoxing Central Hospital, The Central Affiliated Hospital of Shaoxing University, Shaoxing, Zhejiang, China; ^2^ Department of Orthopedics, Shaoxing Central Hospital, The Central Affiliated Hospital of Shaoxing University, Shaoxing, Zhejiang, China; ^3^ Department of Surgery, Shaoxing People’s Hospital, Shaoxing, Zhejiang, China; ^4^ Department of Pain, Shaoxing People’s Hospital, Shaoxing, Zhejiang, China

**Keywords:** osteosarcoma, cGAS-STING, treatment target, drug, tumor immunity

## Abstract

This paper explored the novel approach of targeting the cyclic guanosine monophosphate (GMP)-adenosine monophosphate (AMP) synthase-stimulator of interferon genes (cGAS-STING) pathway for the treatment of osteosarcoma (OS). Osteosarcoma is a common malignancy in adolescents. Most patients die from lung metastasis. It reviewed the epidemiology and pathological characteristics of OS, highlighting its highly malignant nature and tendency for pulmonary metastasis, underscoring the importance of identifying new therapeutic targets. The cGAS-STING pathway was closely associated with the malignant biological behaviors of OS cells, suggesting that targeting this pathway could be a promising therapeutic strategy. Currently, research on the role of the cGAS-STING pathway in OS treatment has been limited, and the underlying mechanisms remain unclear. Therefore, further investigation into the mechanisms of the cGAS-STING pathway in OS and the exploration of therapeutic strategies based on this pathway are of great significance for developing more effective treatments for OS. This paper offered a fresh perspective on the treatment of OS, providing hope for new therapeutic options for OS patients by targeting the cGAS-STING pathway.

## 1 Introduction

**Figure 1:**
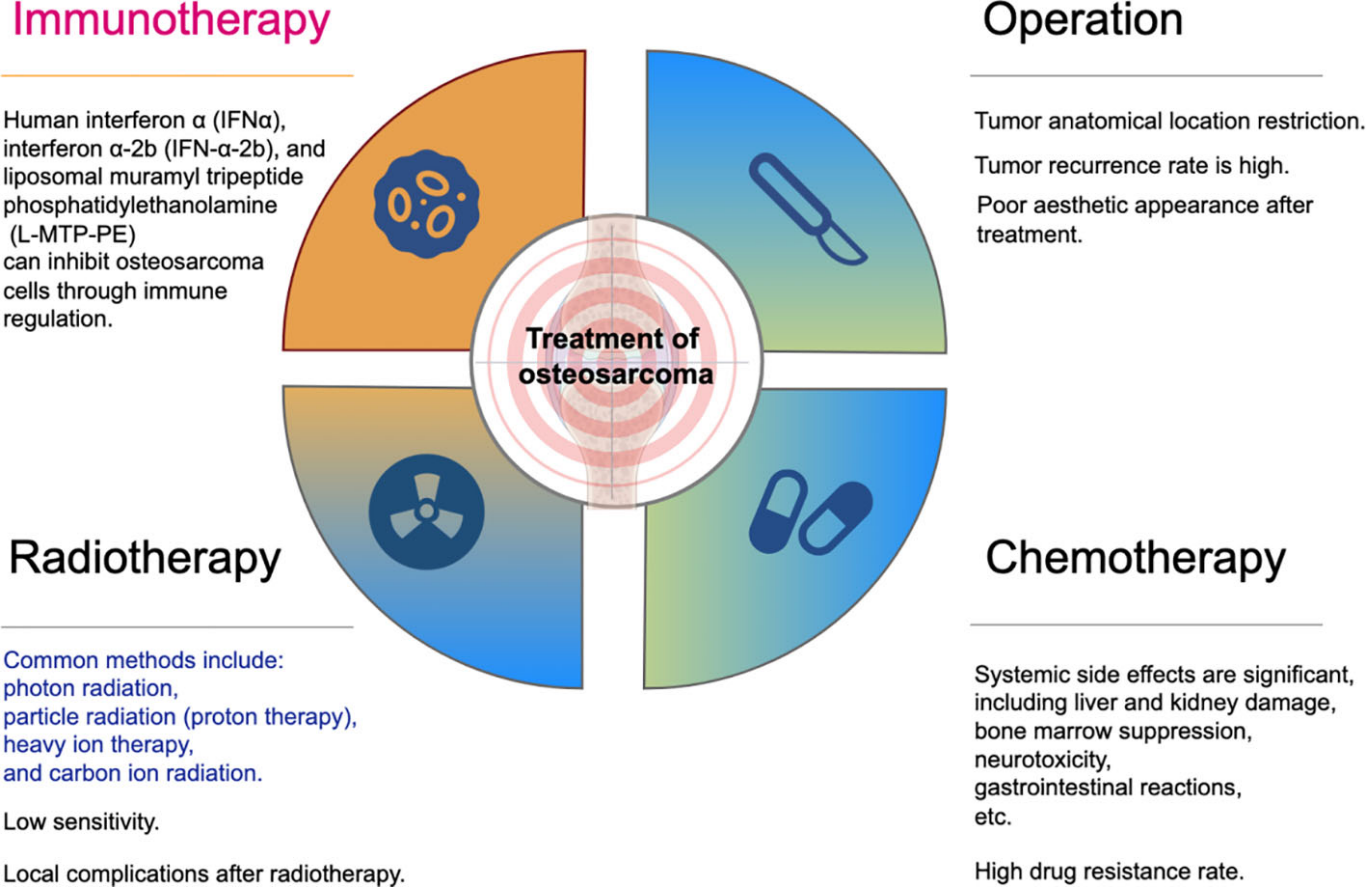
Current treatment of OS.

OS is a malignant bone tumor that primarily affects children and adolescents, particularly those in a rapid growth phase. According to literature reports, the incidence of OS has tripled since 2000, with the highest incidence observed in individuals aged 10 to 24 years, reaching 7.2 cases per million (95% CI: 6.9-7.5) (1). Although the overall incidence is relatively low, its highly malignant nature and tendency for pulmonary metastasis contribute to a high mortality rate, posing a significant threat to the health of adolescents. Current treatment primarily has involved a combination of surgery, chemotherapy, and radiotherapy. Traditional treatments for osteosarcoma include surgical resection and systemic chemotherapy. Surgery is mainly divided into amputation and limb-salvage surgery. Surgery means complete removal of the tumor. Amputation requires that the osteotomy plane is at least 5 cm away from the tumor-free boundary. If the lesion cannot be completely removed during limb-salvage surgery, the local recurrence rate can be as high as 25% (2). At best, only 10% of all patients with osteosarcoma can be cured by tumor resection alone, and most develop local recurrence and/or lung metastases months later. Adjuvant systemic chemotherapy can significantly improve a patient’s chance of cure (3–5). Adjuvant systemic chemotherapy includes postoperative chemotherapy used to remove lesions that cannot be completely removed by surgery and preoperative chemotherapy to improve the success rate of limb-sparing surgery and reduce the risk of recurrence, which have significantly improved the 5-year survival rate of patients with osteosarcoma. However, it is impossible to avoid the systemic side effects caused by chemotherapy, including liver and kidney damage, bone marrow suppression, neurotoxicity, gastrointestinal reactions, etc. For example, doxorubicin can cause permanent myocardial damage, and cisplatin can cause high-frequency hearing loss. wait (6–8). Although radiotherapy can be used for patients whose tumors cannot be surgically removed or remain at the resection margin, and for OS patients whose tumors do not respond well to chemotherapy, the actual sensitivity of OS to radiotherapy is not high (9). While these treatments improve survival rates, they also present challenges such as chemotherapy resistance and high recurrence rates, indicating the need for better treatment options and improved patient quality of life (4, 10). Immunotherapy is a hot research direction at present and is considered to be one of the breakthroughs in the treatment of osteosarcoma (11–15). The tumor microenvironment exists as an immune cell network with complex functions that can promote OS growth. Tumor-derived exosomes can drive bone cell behavior and create conditions for tumor cell homing (16). On the other hand, exosomes also widely promote immunosuppression, such as inhibiting the activity of T cells and NK cells, inducing T cell apoptosis, etc., to help osteosarcoma cells escape immune system (17–19). In addition, many factors such as specific proteins in OS-derived exosomes, cancer-associated fibroblasts, TGF-β, VEGF, tumor-associated macrophages, etc. have their own roles in the osteosarcoma microenvironment, some of which mediate the downregulation of immune cells, some of which provide support for tumor growth, regulate tumor progression, or affect the immune response (20, 21). A strongly suppressive immune microenvironment is associated with overactivation of multiple immunosuppressive pathways, so there is an urgent need to gain a deeper understanding of the osteosarcoma immune system and use its immune markers to develop targeted immunotherapy (22). The abnormal regulation of the immune system is crucial for the occurrence of OS. During interactions between the bone microenvironment and OS cells, the loss or dysfunction of the fatty acid synthase protein within OS cells allows them to evade immune surveillance, particularly in metastatic environments such as the lungs, which constitutively express fatty acid synthase ligands. It allows tumor cells to bypass the host’s defense mechanisms, significantly reducing the efficacy of immune monitoring and clearance. Additionally, the macrophage migration inhibitory factor in OS activates the RAS/MAPK pathway, further promoting tumor cell escape and invasion (23). The formation of an immunosuppressive microenvironment and chronic inflammation provides a conducive environment for tumor growth. Metastatic cells with osteolytic potential in bone metastases can induce OS cells to produce factors such as parathyroid hormone-related protein, transforming growth factor-beta, or interleukin 11, which interact with the RANKL-RANK pathway between osteoblasts and osteoclasts, stimulating osteoclast activation. Simultaneously, the expression of RANK enhances the invasive ability of tumor cells. In environments with impaired immune function, this increases the risk of pulmonary metastasis, contributing to bone tumor progression. These abnormal immune responses not only exacerbate OS progression but also complicate immunotherapy, highlighting the immune system as a potential target for treatment. Therefore, understanding these abnormal regulatory mechanisms is crucial for developing more effective OS treatment strategies (24). (**Figure 1**) The cGAS-STING pathway is closely related to the regulation of the tumor’s immune microenvironment. In recent years, the cGAS-STING pathway has received increasing attention in the immunotherapy research of osteosarcoma (25, 26). Therefore, we chose cGAS-STING pathway for discussion in this review, although Jordan et al. recently published a review with a similar theme (27). The main focus of these two reviews is different. Our main focus is on the cGAS-STING pathway and its upstream and downstream molecular mechanisms. The paper published by Jordan et al. mainly focuses on the application of nanotechnology in targeting the cGAS-STING pathway.

Traditional treatment methods for osteosarcoma include surgical resection, chemotherapy, and radiotherapy as adjuvant therapies. However, due to limitations such as restricted surgical anatomical locations, high tumor recurrence rates, poor aesthetic outcomes, significant systemic side effects of chemotherapy, and low sensitivity to radiotherapy, the advantages of immunotherapy have come to the fore. Studies indicate that immunological agents such as human interferon α, interferon α-2b and liposomal muramyl tripeptide phosphatidylethanolamine can effectively inhibit or reduce osteosarcoma cells.

The cGAS-STING pathway is a well-studied immune pathway. It activates innate immunological responders(IRs), forming a broadly applicable surveillance mechanism to defend against tissue damage and pathogen invasion (28). The pathway recognizes cytoplasmic double-stranded DNA (dsDNA) and promotes type I interferon (IFN) inflammatory signaling responses, while also influencing processes such as autophagy, cell survival, and senescence. It interacts with other innate immune pathways, regulating responses to infections, inflammatory diseases, and cancer, contributing to the impacts of immunotherapy (29). The cGAS-STING pathway is abnormally activated in various tumors, including hepatocellular carcinoma, acute myeloid leukemia, and OS, and plays a role in their occurrence and development (30–32). In OS, the abnormal activation of this pathway is closely associated with the malignant biological behaviors of tumor cells (25). This paper aimed to explore the mechanisms of the cGAS-STING pathway in OS and identify therapeutic strategies based on this pathway. We outlined the structure and function of the cGAS-STING pathway and its role in innate IRs, and analyzed the abnormal activation of this pathway in OS and its relationship with tumor cell proliferation, invasion, and metastasis. We also discussed therapeutic strategies (including small molecule inhibitors alongside immunotherapy), and the challenges and prospects of targeting this pathway for OS treatment, providing a new perspective on OS treatment.

## 2 Structure and function of cGAS and STING proteins

cGAS is composed of a double-globular domain with two spherical structures connected by a groove. Its C-terminal portion contains a nucleotide transferase domain, which includes a catalytic domain and two DNA binding sites (A and B). DNA binding site A induces conformational changes in the protein, repositioning the catalytic pocket to allow catalysis with ATP and GTP substrates. The unique structure of cGAS enables it to effectively recognize and bind to DNA, cGAMP synthesis, and activate the IRs. STING (stimulator of interferon genes) is a protein composed of four transmembrane helices, a cytoplasmic ligand-binding domain (LBD), and a C-terminal tail. The LBD undergoes conformational changes upon binding to cGAMP, promoting STING oligomerization. cGAS and STING are key proteins in the cGAS-STING pathway, playing important roles in DNA recognition and activation of downstream signaling. cGAS catalyzes cGAMP synthesis, which acts as a second messenger to activate STING. STING then recruits and activates TBK1, initiating downstream signaling that leads to an IR. When dsDNA, whether exogenous or endogenous, is detected in the cytoplasm due to DNA damage or pathogen infection, cGAS catalyzes the synthesis of cGAMP. STING, located on the endoplasmic reticulum, recognizes and binds cGAMP, triggering conformational changes, including a 180° rotation and inward folding of the LBD, promoting STING oligomerization. Activated STING is transported from the endoplasmic reticulum to the Golgi apparatus via specific signaling pathways, where it recruits and activates numerous TBK1 molecules. Upon activation, TBK1 phosphorylates interferon regulatory factor 3 (IRF3) and nuclear factor kappa B transcription factors, promoting their translocation into the nucleus and the expression of IFN-α/β and tumor necrosis factor-alpha genes. These genes enhance innate IRs and initiate adaptive IRs (33–35). In addition to this classical pathway, STING can also mediate autonomous defense functions through gene transcription, with autophagy playing a key role. The activation of STING not only triggers antiviral IRs but also induces cellular “senescence” and eventually leads to cell death. Autophagy, senescence, and apoptosis are crucial mechanisms by which the cGAS-STING pathway combats pathological changes and maintains cellular homeostasis (33, 35, 36).

## 3 Relationship of cGAS-STING pathway with different diseases

The cGAS-STING pathway plays a crucial role in tumor immunology. For example, tumor cells can produce DNA damage, activate the cGAS-STING pathway, and trigger inflammatory responses and cell senescence, thereby inhibiting tumor growth. Additionally, tumor cells can evade immune destruction by degrading cGAS, STING, or TBK1 proteins. Radiation therapy, a traditional cancer treatment, plays an important role in clinical applications. It directly kills tumor cells and also activates the cGAS-STING pathway, regulating downstream signals to improve the effectiveness of cancer treatment. Studies have found that expression levels of genes related to the cGAS-STING pathway are low in non-small cell lung cancer cells. Researchers used the cGAS-STING pathway activator diABZI and the small molecule human polynucleotide kinase/phosphatase inhibitor A12B4C3 (which promotes DNA damage) to enhance the activation of this pathway. The results showed that both diABZI and DNA damage increased the sensitivity of NSCLC cells to radiotherapy by promoting apoptosis, offering a new direction for combining radiotherapy with immunotherapy (37). In liver cancer research, a nanoplatform was used to activate the cGAS-STING pathway and enhance the effectiveness of immunotherapy. This platform uses manganese ions (Mn²^+^) and β-paclitaxel to activate the cGAS-STING pathway, upregulate programmed death-ligand 1 (PD-L1), enhance T cell responses, and inhibit tumor growth and metastasis (38). Another study has demonstrated the potential of the cGAS-STING pathway in treating gastric cancer, where metformin can promote the release of downstream inflammatory factors by activating this pathway, enhancing antitumor IRs. The mechanism lies in the fact that metformin inhibits protein kinase B (AKT) phosphorylation, downregulating the expression of the transcription factor sex-determining region Y-box 2 (SOX2). SOX2 downregulation inhibits the AKT signaling pathway, thereby activating the cGAS-STING pathway (39). While cell senescence has a double-edged role in cancer, it can inhibit tumor progression by halting the cell cycle and enhancing immune surveillance (40). In breast cancer treatment, nanomaterials have been used to activate the cGAS-STING pathway, producing hydrogen sulfide and carbon monoxide gases. These gases induce mitochondrial dysfunction and tumor cell apoptosis while stimulating inflammation and dendritic cell maturation, ultimately promoting antitumor IRs and inhibiting the growth and metastasis of breast cancer (41). Many studies also suggest that various factors in multiple cancers activate the cGAS-STING pathway to enhance antitumor IRs (42).

Immune checkpoints are molecules that interact between immune cells, and under normal conditions, they regulate IRs and prevent excessive damage to the body’s own tissues (43). However, in the tumor microenvironment (TME), tumor cells exploit immune checkpoints to suppress immune cell activity, evading immune surveillance and promoting tumor growth and metastasis. Immune checkpoint blockade (ICB) therapy targets these immune checkpoints, relieving their inhibitory effects, reactivating immune cells, and enhancing antitumor IRs (44–46). The cGAS-STING pathway, a cytoplasmic DNA sensor, can recognize dsDNA in the cytoplasm and activate innate IRs. Studies have found that the cGAS-STING pathway works synergistically with ICB therapy to enhance antitumor IRs. This is because the ataxia telangiectasia mutated protein, a key factor in DNA damage repair, is absent, leading to increased cytoplasmic DNA levels, which activate the cGAS-STING pathway, enhancing the efficacy of ICB therapy (47).

The cGAS-STING pathway plays a key role in infectious diseases. During infection, pathogen DNA is released into the cytoplasm, where cGAS recognizes and binds to this DNA, catalyzing the synthesis of cGAMP and activating STING protein. STING then initiates downstream signaling, activating TBK1 and IRF3, which induce the production of type I IFNs and inflammatory factors. These factors activate immune cells and trigger inflammatory and adaptive IRs to eliminate pathogens. The cGAS-STING pathway plays an important role in various infectious diseases. For example, during Kaposi’s sarcoma-associated herpesvirus infection, the pathway is activated, inhibiting viral replication and enhancing IRs. The viral IFN regulatory factor 1 protein encoded by KSHV can inhibit STING-mediated DNA sensing, affecting viral replication and host IRs (48). Another study found that human glial cells express high levels of cGAS and downstream STING proteins in both resting and activated states, improving their ability to recognize viral DNA, activate IRF3, and express IFN-β mRNA, enhancing antiviral capacity (49). In dengue virus infections, which involve an RNA virus, the cGAS-STING pathway is activated, triggering antiviral IRs. Dengue virus can activate this pathway through mechanisms such as IL-1β-induced mitochondrial DNA (mtDNA) release and direct activation of cGAS. However, dengue virus infection can also inhibit the cGAS-STING pathway, such as through the degradation of cGAS or inhibition of STING signal transduction, demonstrating the dual nature of the pathway in viral infections (50–52). In bacterial infections, the cGAS-STING pathway plays an important role in host defense. It recognizes bacterial DNA and regulates innate IRs through a cascade of reactions. In a respiratory tract infection model, STING knockout mice exhibited higher bacterial loads, indicating the cGAS-STING pathway’s importance in controlling Brucella infection (53). Similarly, following Mycobacterium bovis infection, the cGAS-STING pathway promotes the maturation and activation of DCs and enhances CD4+ T cell proliferation, bolstering adaptive IRs to clear the infection (54). In fungal infections, the cGAS-STING pathway is a key pattern recognition receptor in host defense, particularly in corneal epithelial cells. Fungal DNA or RNA hybrids are recognized by cGAS in the cytoplasm, triggering the pathway, promoting IFNs and inflammatory cytokines, and initiating IRs to clear fungal pathogens. Additionally, the pathway induces autophagic flux by enhancing the formation of microtubule-associated protein 1 light chain 3-, participating in clearing intracellular DNA and viruses, which helps the host fight fungal infections (55). Therefore, the cGAS-STING pathway plays an essential role in various infectious diseases, acting as the body’s first line of defense against pathogen invasion. By activating this pathway, the body effectively responds to diverse infectious challenges and achieves self-protection (36, 56–58) (**Table 1**).

**Table 1:** The role of cGAS-STING signaling pathway in different types of diseases.

Disease Type	Disease	Effects of cGAS-STING signaling pathway	Reference
Cance	Lung cancer	diABZI and promotion of DNA damage activate the cGAS-STING pathway and increase the sensitivity of NSCLC cells to radiotherapy.	(37)
	Liver cancer	It enhances T cell responses and inhibits tumor growth and metastasis by upregulating programmed death-ligand 1.	(38)
	Gastric cancer	It promotes the release of downstream inflammatory factors and enhance anti-tumor immune response	(39)
	Breast cancer	It promotes anti-tumor immune response and inhibit the growth and metastasis of breast cancer.	(41)
infection	Kaposi sarcoma-associated herpes virus	It inhibits viral replication and enhance immune response	(48)
	Dengue virus	Activation of the cGAS-STING pathway can induce cell damage and apoptosis	(50)
	Brucella	STING knockout mouse model of respiratory infection exhibits higher bacterial load	(53)
	Mycobacterium bovis	It enhances adaptive immune responses to clear and fight infection	(54)
	Aspergillus fumigatus	It promotes the expression of IFNs and other inflammatory cytokines, triggering host immune responses and clearing fungal pathogens.	(55)

## 4 Role of cGAS-STING pathway in OS

The TME is a highly dynamic and evolving system, making accurate prediction challenging. The TME functions like nutrient-rich soil, providing nourishment for tumor cell proliferation while restricting anti-tumor immunity (59). The cGAS-STING pathway supports tumor survival and proliferation by promoting the formation of an immunosuppressive TME (60). Zhang et al. (61) constructed a Cox proportional hazards regression model and found that high expression of C–C motif chemokine ligand 5 was associated with a favorable prognosis in children with OS. The mechanism involves high expression of C–C motif chemokine ligand 5 significantly increasing the infiltration levels of macrophages (M0, M1), CD8+ T cells, and regulatory T cells in tumor tissues. Henrich et al. (62) developed a model for Ewing’s sarcoma and discovered that ubiquitin-specific protease 6 significantly enhanced the infiltration of macrophages (F4/80+), DCs (CD11c+), and myeloid cells (CD11b+) in primary Ewing’s sarcoma tumors through the synergistic effect of inducing chemokines such as C–X–C motif chemokine ligand 10, resulting in a significant improvement in overall survival rates. C–C motif chemokine ligand 5 and 10 can promote the infiltration of DCs and immune effector cells in OS. Radiotherapy can elevate the expression levels of C–C motif chemokine ligand 5 and 10 (63); however, this effect is not universally present in all cells. In U2OS OS cells with low STING expression, this effect is not observed. The use of STING agonists can alter this phenomenon (63). STING signaling is essential for radiation-induced expression of C–C motif chemokine ligand 5 and 10 in OS cells. Therefore, enhancing STING signaling may be beneficial for OS treatment. Sodium-glucose cotransporter 2 is a mediator of epithelial glucose transport and is highly expressed in many tumor types. Inhibition of Sodium-glucose cotransporter 2 can exert anticancer effects in various tumors, including HCC, pancreatic cancer, prostate cancer, colorectal cancer, lung cancer, and breast cancer (64–67). Wei et al. found that Sodium-glucose cotransporter 2 inhibitors can upregulate the cGAS-STING pathway and induce immune cell infiltration. Furthermore, the combination of Sodium-glucose cotransporter 2 inhibitors and the STING agonist 2’3’-cGAMP exhibited synergistic antitumor effects in OS (32). However, whether STING has an antitumor effect in tumor treatment remains controversial (68). Inducing apoptosis is a commonly employed antitumor strategy. The IFN gamma inducible protein 16/p53 pathway is a mechanism of cell apoptosis. Studies have shown that STING can promote the degradation of IFN gamma inducible protein 16. Additionally, overexpression of STING inhibits p53 serine 392 phosphorylation, p53 transcriptional activity, p53 target gene expression, and p53-dependent mitochondrial depolarization and apoptosis (69). Therefore, further research is needed to identify the therapeutic targets of the cGAS-STING pathway in OS.

The biological characteristics of OS have driven a surge of interest in developing new antitumor drugs based on tissue engineering. One promising approach involves targeting reactive oxygen species (ROS), which are by-products of cellular oxygen metabolism, such as superoxide anion, hydrogen peroxide, hydroxyl radical, and nitric oxide. These ROS are mainly generated by complexes I and III of the mitochondrial inner membrane respiratory chain and by nicotinamide adenine dinucleotide phosphate oxidase on the cell membrane. While ROS play a crucial role in cellular signaling and homeostasis, they are also associated with the occurrence and progression of cancer. Under normal conditions, cells maintain a balance in ROS levels via the antioxidant defense system. However, when this balance is disrupted, excessive ROS can lead to DNA damage, genomic instability, and carcinogenic mutations, thus promoting cancer progression (70, 71). In tumor cells, this imbalance is often caused by mitochondrial dysfunction, which leads to an impaired electron transport chain, reduced mitochondrial membrane potential, increased nicotinamide adenine dinucleotide phosphate oxidase expression, and iron metabolism disorders. These factors, coupled with the excessive proliferation of tumor cells and reduced antioxidant enzyme activity, contribute to elevated intracellular ROS levels (72). Based on this mechanism, Xiang et al. developed composite nanoparticles composed of ROS-sensitive amphiphilic polymers designed to activate the cGAS-STING pathway. These nanoparticles dissociate within the cell in response to ROS, releasing Pt(IV)-C12 and NLG919. The former induces DNA damage, which activates the cGAS-STING pathway and promotes the infiltration of CD8+ T cells into the TME, while the latter enhances the activity of these CD8+ T cells, boosting the IR against cancer cells. For patients with inoperable or metastatic OS, radiotherapy is a critical treatment method. However, in some TMEs with strong immunosuppression, low-dose radiotherapy can lead to radio resistance in tumor cells (73, 74), whereas high-dose radiotherapy may cause damage to immune cells and healthy tissues. Experimental studies have shown that a Ta-Zr co-doped metal-organic framework has significant synergistic effects in enhancing radiotherapy sensitization, photodynamic therapy, and immunotherapy in OS cells. The radiotherapy-radiotherapy dynamic therapy effect mediated by Ta-Zr co-doped metal-organic framework induces DNA damage, which activates the cGAS-STING pathway, stimulating antitumor IRs. Notably, PD-L1 expression stimulated by the cGAS-STING pathway in the Ta-Zr co-doped metal-organic framework+X-ray group was twice that of the control and unirradiated Ta-Zr co-doped metal-organic framework groups, promoting a stronger antitumor IR in radiotherapy.

## 5 Therapeutic strategies targeting the cGAS-STING pathway

Given the significant potential of cGAS-STING pathway in tumor treatment, many researchers are actively investigating therapies that target this pathway. Here, we summarized recent findings related to drug treatments aimed at modulating cGAS-STING activity across various cancers.

Several commonly used chemotherapeutic drugs have been shown to activate the cGAS-STING pathway, contributing to their antitumor effects. For instance, Hu et al. (75) demonstrated *in vitro* that paclitaxel could activate cGAS signaling in certain triple-negative breast cancer cell lines, inducing the polarization of macrophages toward the M1 phenotype and recruiting lymphocytes to the TME, and improving patient survival when combined with other treatments. However, this lymphocyte infiltration does not occur in all triple-negative breast cancer cases, and corresponding *in vivo* studies are lacking. Future research could address this gap and explore the variability in response to paclitaxel. In another study, Li et al. (76) found that arsenic trioxide-induced mitochondrial damage could activate the cGAS-STING pathway in hepatocellular carcinoma cells, enhancing the expression of IFNs. At the same time, STING activation was also associated with increased expression of the immune checkpoint protein PD-L1 in tumor cells. arsenic trioxide treatment improved antitumor immunity and immunogenicity in arsenic trioxide-sensitive hepatocellular carcinoma cells, although arsenic trioxide-insensitive hepatocellular carcinoma cells showed limited response. Future research could focus on improving arsenic trioxide sensitivity in these resistant cells. Notably, when arsenic trioxide-pretreated tumor cells were injected into mice, the treatment also showed both preventive and therapeutic effects, significantly reducing tumor growth, providing a new avenue for the development of hepatocellular carcinoma vaccines. In addition to chemotherapeutic agents, some drugs traditionally used for non-cancer treatments have also been found to activate the cGAS-STING pathway in tumors. Metformin, a classic drug for type 2 diabetes, has been found in recent years to have antitumor effects in several cancers, including lung, pancreatic, breast, prostate, and colon cancer (77–79). Most of these antitumor mechanisms were found to be independent of cGAS-STING pathway. However, Shen et al. (39) found that metformin could activate the cGAS-STING pathway via the SOX2/AKT axis in gastric cancer cells, promoting the release of inflammatory factors and enhancing the effectiveness of immunotherapy. This raises the question of whether metformin may exert similar cGAS-STING-mediated effects in the treatment of other tumors, warranting further investigation.

**Figure 2:**
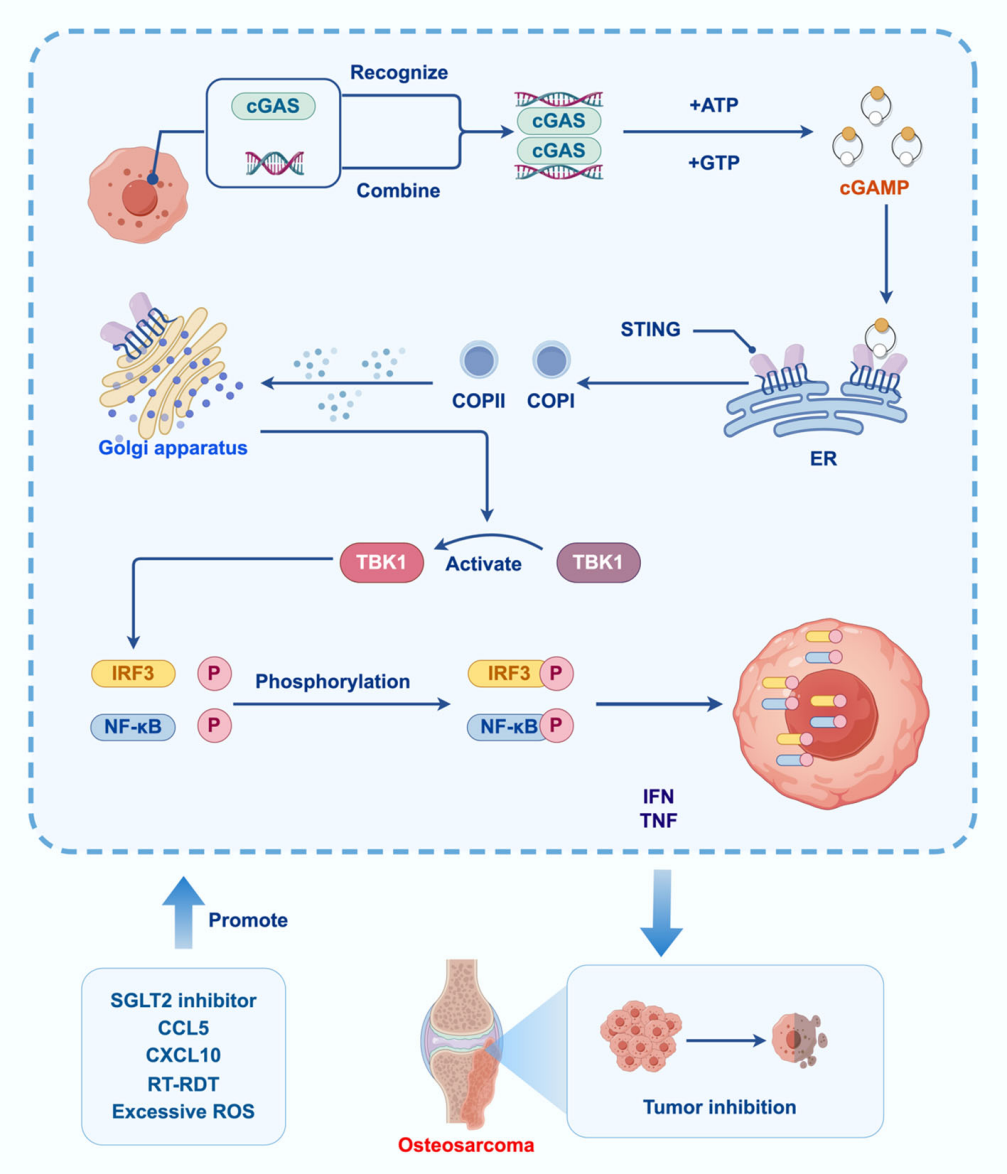
Effect of cGAS-STING signaling pathway in OS.

Lovastatin, an inhibitor of 3-hydroxy-3-methylglutaryl coenzyme A reductase, is widely used to treat hyperlipidemia but also shows promise in cancer treatment. Huang et al. (80) demonstrated that lovastatin activated the cGAS-STING pathway by increasing the abundance of mtDNA in the cytoplasm through mitochondrial damage. This activation resulted in growth inhibition and apoptosis across various cancer cell types. In HCT116 xenograft tumor models, lovastatin effectively inhibited tumor growth via the cGAS-STING pathway. Knocking out cGAS or STING diminished its antitumor effects. Nonsteroidal anti-inflammatory drugs also play a role in tumor treatment. Kosaka et al. (81) found that celecoxib, a selective cyclooxygenase-2 inhibitor, enhanced the antitumor effect of the STING agonist cGAMP in a T cell-dependent manner, inducing systemic tumor-specific IRs in mouse models. Additionally, Zhu et al. (82) showed that aspirin, a targeted drug for inhibiting cGAS-STING signaling, significantly improved asymptomatic orchitis induced by airborne particulate matter. Tumor immunomodulators have been integrated into cGAS-STING targeted therapy. Anlotinib, effective against various tumors such as hepatocellular carcinoma, renal cell carcinoma, and non-small cell lung cancers, glioblastoma, refractory metastatic cervical cancer, and refractory epithelial ovarian cancer, has been shown to enhance tumor control and improve long-term survival (83–88). Yuan et al. (89) established a gastric cancer mouse model and found that anlotinib treatment reduced cell proliferation and invasion by activating the cGAS-STING/IFN-β pathway. Nanotechnology-based targeted therapies focusing on cGAS-STING are gaining attention. Mn²^+^ can enhance antitumor IRs by activating the cGAS-STING pathway (90, 91). Excessive zinc ions (Zn²^+^), which can induce mutant p53 prevalent in many cancers, may relieve inhibition of the cGAS-STING pathway and lead to tumor immunosuppression (92, 93). To harness the synergistic effects of Mn²^+^ and Zn²^+^, Sun et al. (94) constructed MnO_2_-modified zeolitic imidazolate framework 8 nanoparticles, which not only deliver individual ions but also provide dsDNA for the activation of cGAS-STING pathway, enhancing cGAS-STING-mediated antitumor immunotherapy. Fang et al. (95) constructed a manganese-based nanosystem that activates the cGAS-STING pathway to promote the maturation of DCs and enhance the infiltration of cytotoxic T lymphocytes, thereby increasing the sensitivity to ICB immunotherapy. Additionally, Li et al. (96) created an iron-based metal-organic framework nanoparticle reactor loaded with dihydroartemisinin that induces DNA damage to activate the cGAS-STING pathway, facilitating the binding of STING and IRF3 and promoting anticancer immunotherapy. Scutellarein, a natural compound isolated from schisandra lignans, has also been shown to activate the cGAS-STING pathway, inhibiting hepatitis B virus replication and chronic hepatitis B (97). Yang et al. (98) further found that SC reduced tumor growth by enhancing type I IFN responses in a cGAS-STING pathway-dependent manner. Moreover, when combined with platinum chemotherapy, Scutellarein enhanced the antitumor effects of cisplatin while mitigating side effects. These findings highlight the potential of cGAS-STING-targeted therapies and nanotechnology in cancer treatment **Figure 2**, **Table 2**.

**Table 2:** Tumor treatment of drugs targeting the cGAS-STING pathway.

Drug name	dose	cell lines	*In Vivo* Studies	Effects	References
Metformin	5 mmol/L	BGC823, AGS, SGC7901	none	Metformin exerts an anti-tumor effect in gastric cancer immunotherapy by inhibiting SOX2/AKT activation of the cGAS/STING signaling pathway.	(39)
Anlotinib	8μM	AGS,HS746T	Subcutaneous tumor model	Anlotinib may inhibit the proliferation, migration, and immune evasion of gastric cancer cells by activating the cGAS-STING/IFN-β pathway.	(89)
Schisandrin C	30 mg/kg	MC38,4T1	Subcutaneous tumor model	Schisandrin C reduces tumor growth by enhancing type I IFN response in a cGAS-STING-dependent manner.	(98)
Lovastatin	10 μM	HCT116,HEK293T	Subcutaneous tumor model	Lovastatin triggers accumulation of mitochondrial DNA in the cytoplasm through oxidative mitochondrial DNA damage, thus activating the cGAS-STING pathway in CRC.	(80)
Arsenic trioxide	3 mg/kg	Hepa1-6,Huh7,MHCC97H,Hep G2, Hep 3B,H22	Subcutaneous tumor model	Arsenic trioxide activates the cGAS/STING/IFN cascade through induction of mitochondrial damage and mtDNA release, exerting an immunostimulatory effect.	(76)
Paclitaxel	10nM	MDA-MB-231,BT-549,MDA-MB-468,MDA-MB-436,MDA-MB-453,Hs578T, HCC1806	none	Paclitaxel induces macrophage polarization to M1 phenotype in a cGAS-dependent manner, and may help with lymphocyte recruitment in some TNBC samples and better survival in patients receiving combination therapy.	(75)
Celecoxib	200 ppm	4T1-Luc RRID, CVCL_J239, E0771 RRID: CVCL_GR23,CT26	Orthotopic tumor model	Combination treatment with cGAMP and celecoxib significantly inhibits tumor growth through T cell-mediated and STING signaling responses.	(81)

The DNA binding site of cGAS A induces conformational changes in the protein, repositioning the catalytic pocket to allow catalysis with ATP and GTP substrates. The LBD undergoes conformational changes upon binding to cGAMP, promoting STING oligomerization. cGAS catalyzes cGAMP synthesis, which acts as a second messenger to activate STING. STING then recruits and activates TBK1, initiating downstream signaling that leads to an IR. Then TBK1 phosphorylates IRF3 and nuclear factor kappa B transcription factors, promoting their translocation into the nucleus and the expression of IFN-α/β and tumor necrosis factor-alpha genes. Activation of the cGAS-STING signaling pathway leads to osteosarcoma. inhibitory effect. SGLT2 inhibitors, CCL5, CXCL10, RT-RDT, ROS and other factors affect the activation of the cGAS-STING signaling pathway.

## 6 Clinical research

The cGAS-STING signaling pathway is a popular molecular mechanism in recent years. There are few clinical studies related to the cGAS-STING signaling pathway. The cGAS-STING signaling pathway is of great significance in the treatment of tumors. Eribulin is a regulator of the cGAS-STING signaling pathway that can improve the tumor microenvironment. Candace et al. found that the combination of Eribulin and pembrolizumab in metastatic soft tissue sarcoma can achieve better therapeutic effects in liposarcomas and angiosarcomas, and serum IFNα and IL4 levels are associated with clinical benefits (99). Manganese is necessary for cGAS-STING to defend against cytoplasmic dsDNA (100). Lv et al. found in a phase I clinical trial that manganese and anti-PD-1 antibodies were used in combination in patients with a variety of metastatic solid tumors. The results showed that the combined application showed promising efficacy, exhibiting type I IFN induction, manageable safety and revived responses to immunotherapy (90). Combining activators of the cGAS-STING signaling pathway has advantages for tumor treatment. In addition to research on tumors, the cGAS-STING signaling pathway has also been clinically studied in diseases such as anemia and infection (101, 102). There are no reports on clinical studies of the cGAS-STING signaling pathway in osteosarcoma.

## 7 Summary and outlook

The cGAS-STING pathway is emerging as a crucial component in tumor immunology, particularly in OS. Research has shown that this pathway is abnormally activated in OS, correlating with the malignant biological behaviors of tumor cells. Targeting the cGAS-STING pathway presents a promising new approach for the treatment of OS. Recent studies indicate that various small molecule drugs and nanomaterials aimed at the cGAS-STING pathway may serve as potential therapies for OS. For example, SGLT2 inhibitors can upregulate the cGAS-STING pathway and induce immune cell infiltration, while Mn²^+^ can activate the cGAS-STING pathway *in vivo*, promoting antitumor IRs. These findings suggest new ideas for developing OS treatment based on the cGAS-STING pathway. However, research on the role of the cGAS-STING pathway in OS treatment remains limited, and the underlying mechanisms are not fully understood. Therefore, further investigation into the mechanisms of cGAS-STING in OS and the exploration of targeted treatment strategies are of great significance for the development of more effective treatment options for OS. While effective therapies for OS are still lacking, the significance of the cGAS-STING pathway in tumor diseases might provide a new perspective for its treatment. In future, targeted therapies based on the cGAS-STING pathway may offer new hope for OS patients.
